# Tube within Tube: Ascaris in Bowel and Biliary-Tract

**DOI:** 10.4269/ajtmh.2010.10-0358

**Published:** 2010-11-05

**Authors:** Ankur Goyal, Shivanand Gamanagatti, Jaganathan Sriram

**Affiliations:** Department of Radiodiagnosis, All India Institute of Medical Sciences, New Delhi, India

## Abstract

*Ascaris lumbricoides* is one of the most common human helminthic diseases worldwide. On ultrasound, it is seen as linear non-shadowing echogenic structures with target appearance in cross section, and the live worm may show writhing movements in real time. On barium meal follow through, it appears as radiolucent tubular filling defects within the bowel lumen. Though not sensitive, direct real-time visualization of *Ascaris* on ultrasound is quick, non-invasive, and definitive.

A 20-year-old female, residing in a slum area, presented with diffuse pain in the abdomen and occasional vomiting for 3 days. On examination, she was pale and icteric with no significant abdominal findings. Her hemoglobin was 8.5 gm/dL, total bilirubin 3.6 mg/dL, and conjugated bilirubin 2.8 mg/dL. On abdominal ultrasound, linear tubular structures were seen within the small bowel (Figure 1, arrowheads), with parallel echogenic walls and thin echogenic interfaces internally. These structures were seen to exhibit writhing movements in real time. Similar structures were seen in the common bile duct (Figure 2, arrows) and had target/bull's eye appearance in cross section (Figure 2, arrowheads). No posterior acoustic shadowing was seen. There was minimal dilatation of bilateral intra-hepatic biliary radicles. Diagnosis of ascaris infestation was made and subsequently done barium meal follow through showed multiple tubular filling defects in the small bowel (Figure 3, arrowheads). After endoscopic extraction of the worm from the common bile duct, the patient was started on albendazole. No complications (cholangitis or pancreatitis) were seen on follow-up.

**Figure 1.**
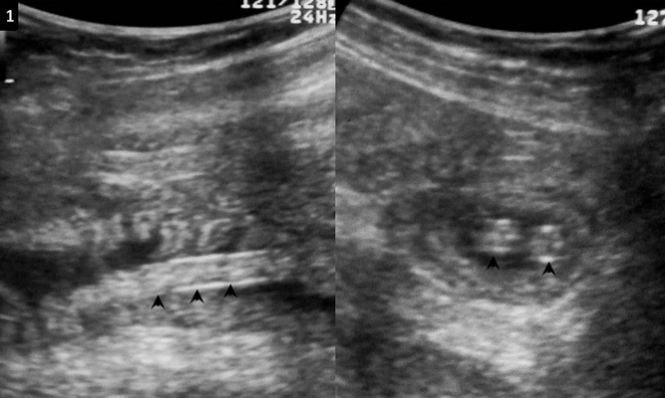
Ultrasound image of small bowel showing *Ascaris* in longitudinal and transverse views.

**Figure 2.**
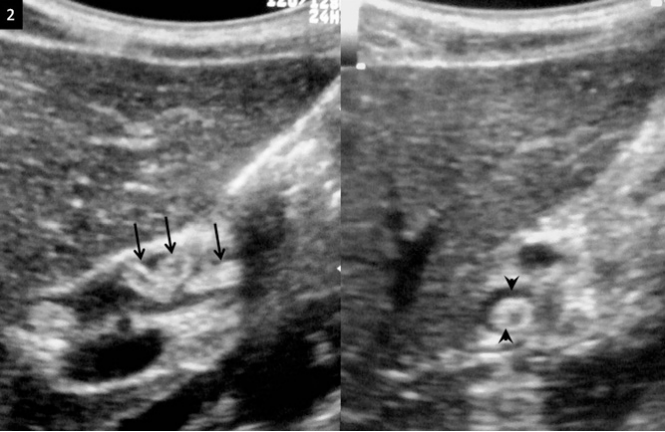
Ultrasound image of common bile duct showing *Ascaris* as tubular structure within the lumen and target sign on cross-section.

**Figure 3.**
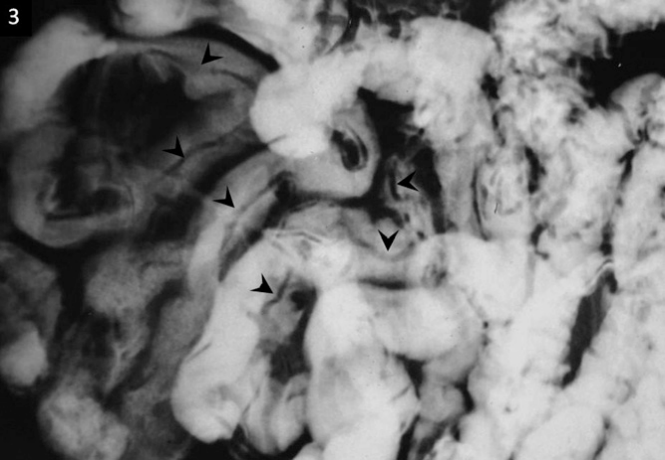
Barium meal follow through image showing *Ascaris* as multiple linear radiolucent filling defects within the small bowel.

*Ascaris lumbricoides* is one of the most common human helminthic diseases worldwide. Ultrasonographic findings depend on the worm's site of infestation, orientation to, and resolution of the transducer probe, the presence or absence of fluid around the worm, the part of the worm imaged, and whether the worm is dead or alive.^1^ Though not sensitive, direct real-time visualization of *Ascaris* on ultrasound is quick, non-invasive, and definitive.
